# A model of long-term survival following adjuvant therapy for stage 2 breast cancer.

**DOI:** 10.1038/bjc.1993.498

**Published:** 1993-12

**Authors:** J. W. Gamel, R. L. Vogel

**Affiliations:** Veterans Administration Medical Center, Louisville, KY.

## Abstract

Following adjuvant therapy for breast cancer, some patients will die of this tumour while the remainder will die of other causes. Deaths from breast cancer tend to follow a lognormal distribution, while deaths from other causes can be approximated by national demographic data. By combining these two survival models, we have generated an age-specific method for estimating the impact of treatment on overall long-term survival. Treatment was designed to operate by one of two mechanisms: an increase in cured fraction, or an increase in median tumour-related survival time among uncured patients. This analysis revealed that, for young and middle-aged patients, an increase in cured fraction has substantially greater long-term clinical impact than an increase in median survival time. Unfortunately, the non-parametric tests traditionally used in prospective clinical trials cannot distinguish between these two mechanisms of action.


Br. J. Cancer (1993), 68, 1167-1170 © Macmillan Press Ltd., 1993

### A model of long-term survival following adjuvant therapy for stage 2 breast cancer

J.W. Gamel[1] & R.L. Vogel[2]

[1]Veterans Administration Medical Center and the Department of Ophthalmology and Visual Sciences; University of Louisville School of Medicine; and [2]The Department of Family Practice, Medical Center of Central Georgia, Macon, Georgia, USA.

**Summary** Following adjuvant therapy for breast cancer, some patients will die of this tumour while the remainder will die of other causes. Deaths from breast cancer tend to follow a lognormal distribution, while deaths from other causes can be approximated by national demographic data. By combining these two survival models, we have generated an age-specific method for estimating the impact of treatment on overall long-term survival. Treatment was designed to operate by one of two mechanisms: an increase in cured fraction, or an increase in median tumour-related survival time among uncured patients. This analysis revealed that, for young and middle-aged patients, an increase in cured fraction has substantially greater long-term clinical impact than an increase in median survival time. Unfortunately, the non-parametric tests traditionally used in prospective clinical trials cannot distinguish between these two mechanisms of action.

In our treatment of patients with cancer, we cannot hope to prolong life forever. Even those patients who are cured of their tumour will eventually die from other causes. The best that we can hope for is to increase survival rate beyond the level that would have occurred without treatment. By this logic, it would seem reasonable to judge the relative effectiveness of treatment by comparing survival rates at a specific endpoint following diagnosis.

Indeed, such comparison is the express purpose of modern prospective, randomised clinical trials. Since these trials demand so much of patients and of society at large, we must perform our comparisons with the most appropriate statistical model. The current 'gold standard' for clinical trials includes a variety of non-parametric methods, such as the log rank test (Peto *et al.*, 1976 and 1977). These methods are cherished above all others because their derivations incorporate minimal assumptions. Such paucity of assumptions is achieved by excluding from the model those parameters that govern tumour-related survival – i.e., likelihood of cure and median tumour-related survival time among uncured patients.

It is important to note, however, that cancer treatment is fundamentally a parametric process: Effective therapy must increase rate of cure, increase time to death from tumour among uncured patients, or achieve a combination of these effects. Thus if we wish to measure precisely the long-term impact of treatment, we must consider the impact of therapy on cured fraction and median tumour-related survival time.

Unfortunately, the survival rate at a specific endpoint is not always an accurate measure of these two parameters. An initial advantage in survival rate at five years will vanish with progressive follow-up if the two treatment groups have the same cured fraction. An even more dramatic disparity between short-term and long-term follow-up is shown in Figure 1, which compares two hypothetical treatment groups. That group with the longest median survival time has a higher initial survival rate, while that group with the largest cured fraction has higher long-term survival rate. Non-parametric analysis would distinguish the best long-term therapy only if performed after the curves cross at 10 years.

Figure 1 represents a worst-case scenario, and such crossing of survival curves may be an unlikely event. We are aware of only one example where such a phenomenon has been observed in a clinical trial (Cuzick *et al.*, 1987). Nevertheless, non-parametric methods could lead to serious errors in the analysis of clinical trials: (i) The therapy we select based on short-term follow-up may enhance only survival time and thus offer no long-term survival benefit. (ii) Alternatively, we might conclude after limited follow-up that there is no difference among treatment groups, when in reality one therapy enhances cured fraction and thus offers a significant long-term advantage. (iii) When treatment groups differ substantially in their median survival times, the assumption of proportional hazards is violated, and this assumption is incorporated in many non-parametric methods (Peto *et al.*, 1976 and 1977).

Given both the ambiguity of survival rate at a specific endpoint and the limitations of non-parametric methods, we might be tempted to consider only the impact of therapy on cured fraction. Unfortunately, this approach also poses a number of problems: (i) The parametric methods used to estimate cured fraction are complex and have little power unless available follow-up extends beyond the median value for tumour-related survival time (Boag, 1949; Gamel *et al.*, 1990). (ii) Emphasising only cured fraction ignores the potential clinical benefit to be obtained from prolonging survival time among uncured patients. (iii) Standard forms of both parametric and non-parametric analysis fail to reflect

**Figure 1** The relative impact of cured fraction and median survival time, as demonstrated by two lognormal tumour-related survival curves. The continuous line represents patients with a cured fraction of 0.27 and a median survival time of 8.2 years, while the broken line represents patients with a cured fraction of 0.50 and a median survival time of 3.4 years.

Correspondence: J.W. Gamel, 301 E. Muhammad Ali Blvd. Louisville, KY 40292, USA.
Received 8 April 1993; and in revised form 20 July 1993.

the important role of patient age at the time of treatment; for example, cured fraction will have its greatest impact on the long-term clinical course of young patients, while older patients, because of impending death from other causes, may enjoy only a limited benefit from any therapeutic modality.

These considerations argue for an index of overall survival that allows for the impact of patient age and incorporates both tumour-related deaths and deaths from other causes. We can apply this index to mathematically generated data to assess the long-term impact of both treatment and age. Such analysis may offer important insight into the limitations of clinical trials for stage 2 breast cancer.

### Materials and methods

*Tumour-related survival*

A survival curve can be generated to represent the likelihood $S_B(t)$ that a patient will not die of breast cancer by time $t$ following treatment:

$$S_B(t) = C + (1 - C)\ P(t), \text{ where} \tag{1}$$

$C$ = cured fraction, and

$$P(t) = 1 - \int_{s=0,t} D(s)\ ds,$$

where $D(s)$ is a function with unit integral that represents the distribution of time to death from breast cancer. Alternatively, for a log-based distribution, the range of integration for $D(s)$ becomes $s = -\infty$ to $s = \log t$ (Boag, 1949).

A number of authors have shown that time to death from many cancers tends to follow a lognormal distribution (Boag, 1949; Mould & Boag, 1975; Mould *et al.*, 1976; Gamel *et al.*, 1990). The most extensive study to date of breast cancer (Rutqvist *et al.*, 1984) incorporated 14,731 patients, 5,252 of whom had stage 2 disease. Using this population, Rutvqist derived lognormal estimates of basic survival parameters, including cured fraction ($C$), mean ($M$) and standard deviation ($S$) of log survival time. For patients with stage 2 disease, these values were $C = 0.27$, $M = 1.22$, $S = 1.04$, with median survival time $\exp\{M\} = 3.4$ years. Since these patients were managed in the era before adjuvant therapy, we assume that these parameters represent the baseline clinical course of 'untreated' patients with stage 2 disease. Using these parameters in the lognormal model, the tumour-related survival rate was 53% at 5 years, 38% at 10 years, and 33% at 15 years after diagnosis.

We simulated therapeutic impact by enhancing either cured fraction or mean log survival time to generate a specific increase in the survival rate at follow-up intervals of 5, 10, and 15 years. At each interval, we used equation (1) to increase the survival rate from breast cancer by 20% or 40% (e.g., to 73 and 93% at 5 years). One of two mechanisms was used to generate this increase:

(i) An increase in $C$ was computed to give the selected increase in tumour-related survival rate at the selected follow-up time, keeping $M$ and $S$ constant at the level observed in 'untreated' patients.
(ii) An increase in $M$ was computed to give the selected increase in tumour-related survival rate at the selected follow-up time, keeping $C$ and $S$ constant.

The analysis just described might vary with the particular function used to represent the distribution of tumour-related survival times. To test for such variation, we considered both the loglogit and Weibull distributions, in addition to the lognormal (Elandt-Johnson & Johnson, 1980). To each lognormal model, both of these alternative models were fitted by the least-squared-error technique over the first 15 years of follow-up. Parallel analysis was then run with all three models. The results of lognormal analysis are shown in Table I, while the comparison of loglogit and Weibull models with the lognormal model is shown in Figure 2.

*Survival from other causes*

To represent mortality from causes other than breast cancer, we used all-cause survival data for American women (all races) in the year 1980 (DHHS, 1985). Since this data base includes a component of women who died from breast cancer, an adjustment was made using mortality from breast cancer for the US population of females (all races) for the years 1978–1983 (SEER, 1984). For each year of age, mortality from breast cancer was subtracted from mortality due to all causes. The resulting function $S_O(a)$ is assumed to represent the likelihood that women of any race will survive all other causes (i.e., all causes excluding breast cancer) to age $a$. For women diagnosed as having breast cancer at age $a$, the likelihood that they will survive all other causes to time $t$ following diagnosis is given by $S_O(a + t)/S_O(a)$.

*Survival from all causes*

To allow for death from both breast cancer and other causes among patients with breast cancer, we constructed an index

**Table I** Median survival time from all causes (MSTAC) as a function of increases in cured fraction (C) or mean log tumour-related survival time (M) produced by treatment of patients with stage 2 breast cancer

| *Duration of follow-up (years)* | *Increased survival[a] (per cent)* | *Age = 35* ↑*C* | *Age = 35* ↑*M* | *Age = 55* ↑*C* | *Age = 55* ↑*M* | *Age = 75* ↑*C* | *Age = 75* ↑*M* |
|---|---|---|---|---|---|---|---|
| 0 | 0 | 6 | 6 | 6 | 6 | 5 | 5 |
| 5 | 20 | 34 | 12 | 18 | 10 | 8 | 7 |
| 5 | 40 | 47 | 27 | 28 | 20 | 12 | 10 |
| 10 | 20 | 22 | 13 | 13 | 12 | 7 | 8 |
| 10 | 40 | 44 | 25 | 25 | 19 | 10 | 10 |
| 15 | 20 | 18 | 16 | 12 | 14 | 7 | 8 |
| 15 | 40 | 42 | 29 | 24 | 21 | 9 | 11 |

[a]Tumour-related survival rate of untreated patients is 53% at 5 years, 38% at 10 years, and 33% at 15 years. Percentages shown in this column represent absolute increases in the tumour-related survival rate produced at each follow-up interval by increasing either C (cured fraction) or M (mean log tumour-related survival time) as the result of hypothetical adjuvant therapy.

**Figure 2** The impact of the specific distribution function used for survival analysis. Median survival time from all causes (MSTAC) was computed with the lognormal model as shown in Table I. These values are plotted against values computed with the loglogit mode (XXX) and the Weibull model (OOO). The straight line represents equivalent values.

that incorporates both sources of mortality. For patient's age $a$ at the time of diagnosis, at time $t$ following diagnosis:

$$N(a,t) = \text{likelihood of surviving all causes until time } t$$
$$= S_B(t)\ [S_O(a+t)/S_O(a)]$$
$$T_{50}(a) = t \text{ such that } N(a,t-1) > 0.50 \text{ and } N(a,t) \leqslant 0.50,$$

Thus $T_{50}(a)$ is the median survival time from all causes (MSTAC) for a patient diagnosed with breast cancer at age $a$ – i.e., that year following diagnosis during which survival rate falls below 50%. Note that there is a distinct $T_{50}(a)$ for each $S_B(t)$ generated as described above. For demonstration purposes, analysis was performed for $a = 35$, 55, and 75 years.

### Results

Figure 1 shows two tumour-related survival curves which have the same value after ten years of follow-up, despite substantial differences in cured fraction. From this figure it is apparent that follow-up at 5 or 10 years is not a reliable index of long-term survival.

Table I shows the differential impact on MSTAC of increases in cured fraction ($C$) versus increases in mean log tumour-related survival time ($M$). The first row represents MSTAC for patients that received no adjuvant treatment. For younger patients, survival rate increases at 5 and 10 years that are due to enhanced $C$ yield greater increases in MSTAC than those increases that are due to enhanced $M$. For example, for 35 year old patients, a 20% increase in the survival rate from breast cancer at 5 years yields a 28 year increase in MSTAC if due to enhanced $C$, but only a 6 year increase if due to enhanced $M$. It is important to note that non-parametric survival methods cannot distinguish between these two mechanisms for enhancing survival rate.

Figure 2 shows variations in values of MSTAC when the loglogit or Weibull functions are substituted for the lognormal function in the model of tumour-related survival. It is apparent from this figure that the function selected has relatively little impact on these values.

### Discussion

As noted in the Introduction above, we rely primarily on controlled clinical trials to select the optimum adjuvant therapy for patients with stage 2 breast cancer. We generally make our selection within 5 to 10 years after treatment, using a non-parametric method to determine which group has the best survival rate. Hidden within this process, however, is an unspoken assumption: That treatment which yields the best short-term survival rate will also yield the best long-term clinical course. Unfortunately, we now have several reasons to call this assumption into question.

(1) An earlier study revealed that, with limited follow-up, an increase in cured fraction is more difficult to detect by the log rank test than a comparable increase in median tumour-related survival time (Gamel *et al.*, 1993).
(2) We cannot rely on differences in the survival rate at an early endpoint to determine which treatment yields the largest cured fraction (Figure 1).
(3) The long-term clinical impact of an early increase in survival rate is highly sensitive to the mechanisms involved; treatment that enhances cured fraction has a substantially greater long-term impact than treatment that enhances median tumour-related survival time. This is especially true for younger patients (Table I). Such sensitivity is largely independent of the particular function selected to represent the tumour-related survival rate (Figure 2).

Taking these findings in concert, we must conclude that the short-term survival rate is a limited measure of long-term clinical course. Fortunately, with increasing follow-up:

(1) The log rank test becomes progressively more sensitive to enhanced cured fraction and less sensitive to enhanced median tumour-related survival time (Gamel *et al.*, 1993).
(2) Tumour-related survival rate becomes a more reliable index of cured fraction (Figure 1).
(3) The long-term clinical impact of a specific increase in survival rate becomes less sensitive to cured fraction (Table I).

Unfortunately, the clinical exigencies of adjuvant therapy argue for the shortest possible follow-up. Trials are very expensive, and we want to offer the best treatment to all patients as soon as possible. In seeking to resolve this dilemma, we should give careful consideration to two important issues:

### *Patient age*

The findings shown in Table I suggest that older patients enjoy less benefit from therapy than younger patients. Furthermore, because of the greater likelihood of death from other causes, older patients provide less follow-up data with which to evaluate therapeutic effect.

### *Duration of follow-up*

We do not have sufficient evidence to justify a radical revision of the protocols for clinical trials. For the foreseeable future, we will continue using non-parametric methods to detect an early separation in survival curves. Nevertheless, these protocols could allow for a 'second look' at some predetermined time (e.g., 5 or 10 years) beyond the traditional 'first look'. Such a second look would demand relatively little additional expenditure – specifically, the money required to maintain follow-up surveillance for the additional time interval. Given the initial investment, and given the long-term costs of an erroneous early conclusion, such a cautionary measure would seem to justify the cost.

This work was supported by a Veterans Administration Merit Review Research Grant Project 001, the Kentucky Lions Eye Foundation, and an unrestricted grant from Research to Prevent Blindness, Inc. (New York, New York).

### References

BOAG, J.W. (1949). Maximum likelihood estimates of the proportion of patients cured by cancer therapy. *J. Royal Stat. Soc.*, **11**, 15–44.

CUZICK, J., STEWART, H., PETO, R. & others (1987). Overview of randomized trials of postoperative adjuvant radiotherapy in breast cancer. *Cancer Treat. Rep.*, **71**, 15–29.

DHHS (DEPARTMENT OF HEALTH AND HUMAN SERVICES) (1985). *US Decennial Life Table for 1979–1981*. National Center for Health Statistics, publication number PHS 85-1150-1: Hyatsville, MD.

ELANDT-JOHNSON, R.C. & JOHNSON, N.L. (1980). *Survival Models and Data Analysis*. John Wiley and Sons: New York.

GAMEL, J.W., MCLEAN, I.W. & ROSENBERG, S.H. (1990). Proportion cured and mean log survival time as functions of tumor size. *Stat. Med.*, **9**, 999–1006.

GAMEL, J.W., VOGEL, R.L. & MCLEAN, I.W. (1993). Assessing the impact of adjuvant therapy on cure rate for stage 2 breast carcinoma. *Br. J. Cancer*, **6**, 115–118.

MOULD, R.F. & BOAG, J.W. (1975). A test of several parametric statistical models for estimating success rate in the treatment of carcinoma cervix uteri. *Br. J. Cancer*, **32**, 529–550.

MOULD, R.F., HEARNDEN, T., PALMER, M. & WHITE, G.C. (1976). Distribution of survival times of 12,000 head and neck cancer patients who died with their disease. *Br. J. Cancer*, **34**, 180–190.

PETO, R., PIKE, M.C., ARMITAGE, P., BRESLOW, N.E., COX, D.R., HOWARD, S.V., MANTEL, N., MCPHERSON, K., PETO, J. & SMITH, P.G. (1976). Design and analysis of randomized clinical trials requiring prolonged observation of each patient: I. Introduction and design. *Br. J. Cancer*, **34**, 585–612.

PETO, R., PIKE, M.C., ARMITAGE, P., BRESLOW, N.E., COX, D.R., HOWARD, S.V., MANTEL, N., MCPHERSON, K., PETO, J. & SMITH, P.G. (1977). Design and analysis of randomized clinical trials requiring prolonged observation of each patient: II. Analysis and examples, *Br. J. Cancer*, **35**, 1–39.

RUTQVIST, L.E., WALLGREN, A. & NILSSON, B. (1984). Is breast cancer a curable disease? A study of 14,731 women with breast cancer from the cancer registry of Norway. *Cancer*, **53**, 1793–1800.

SEER PROGRAM. (1984). Cancer incidence and mortality in the United States 1973–1981. *NIH Publication No. 85-1837*. National Cancer Institute: Washington.


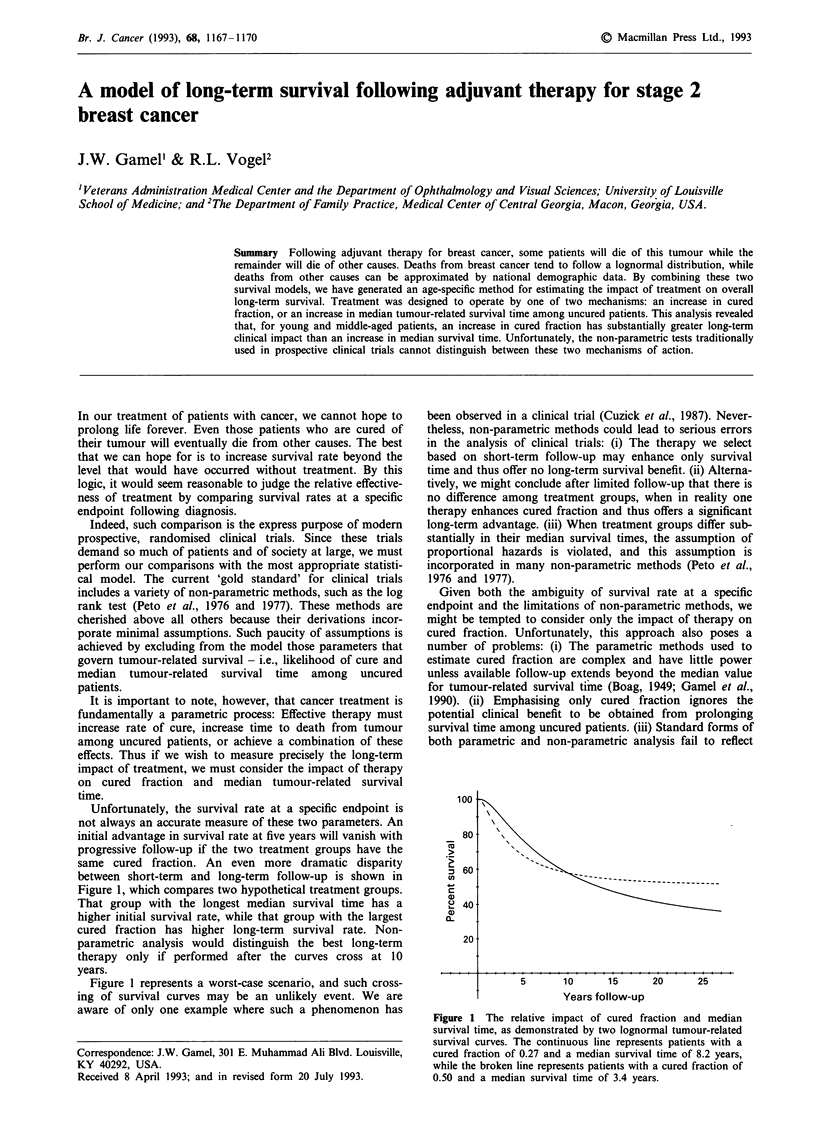

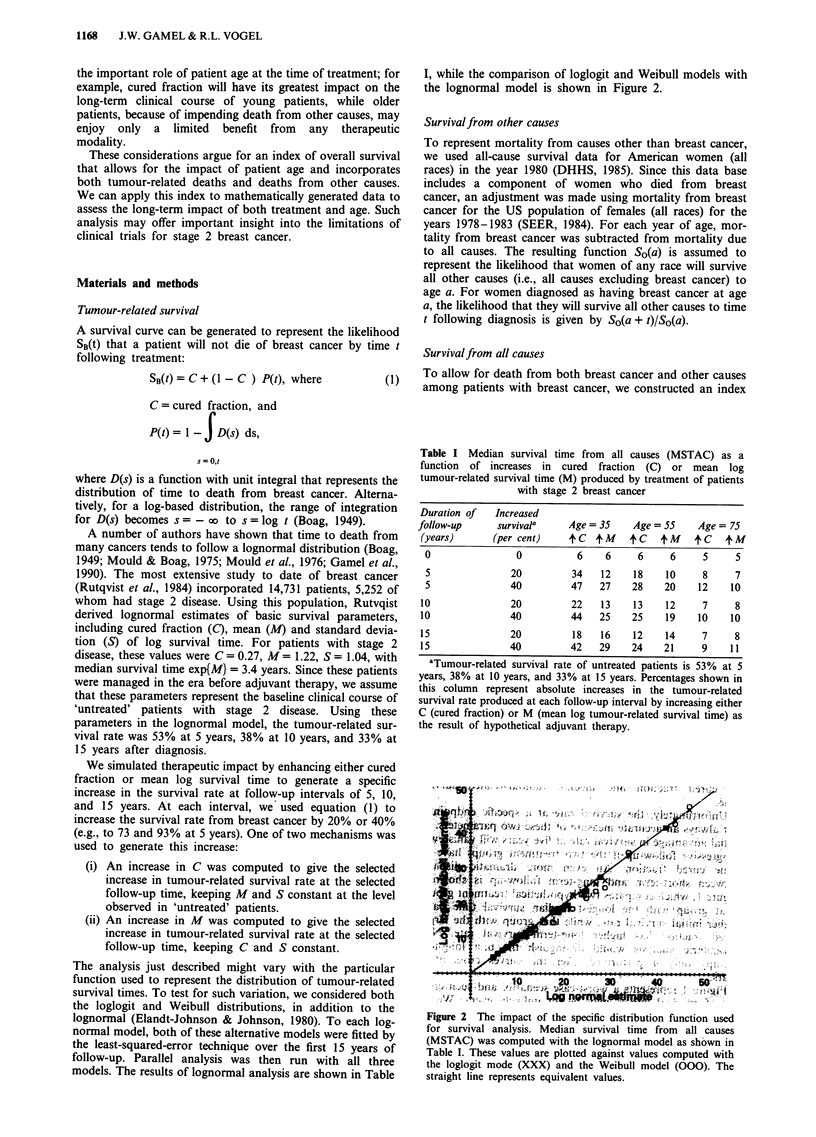

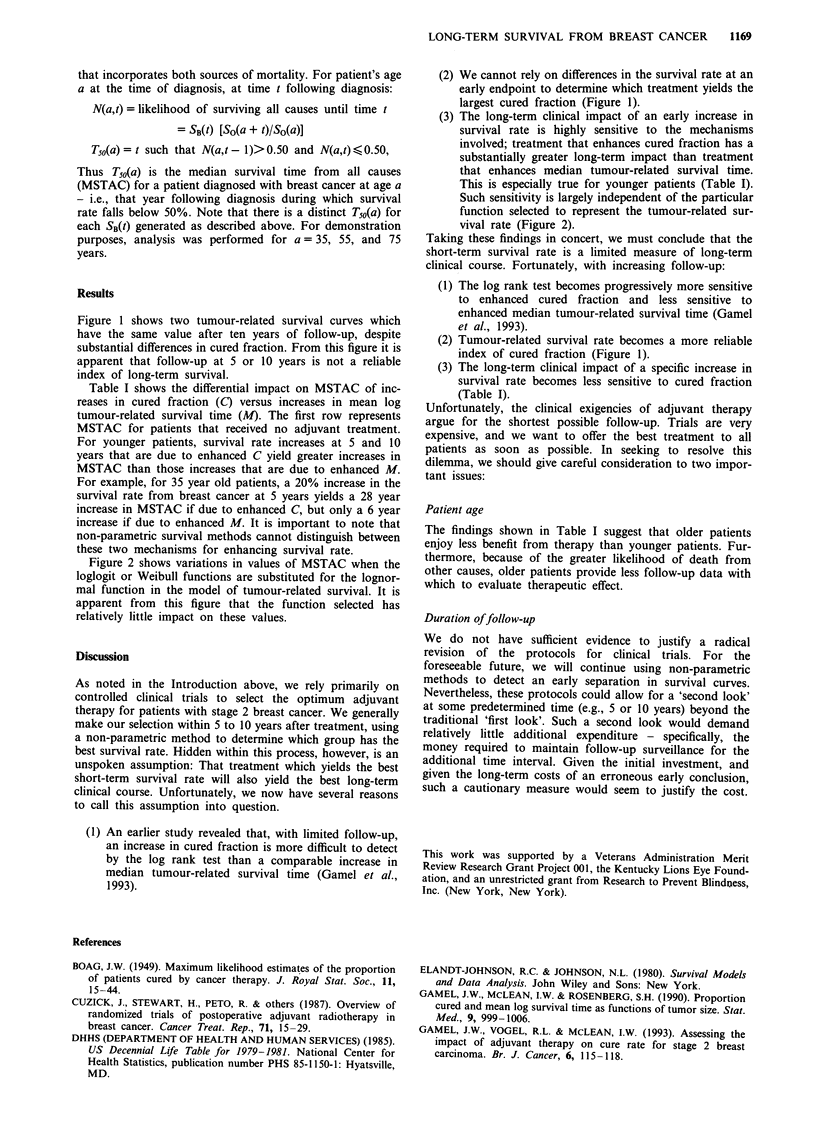

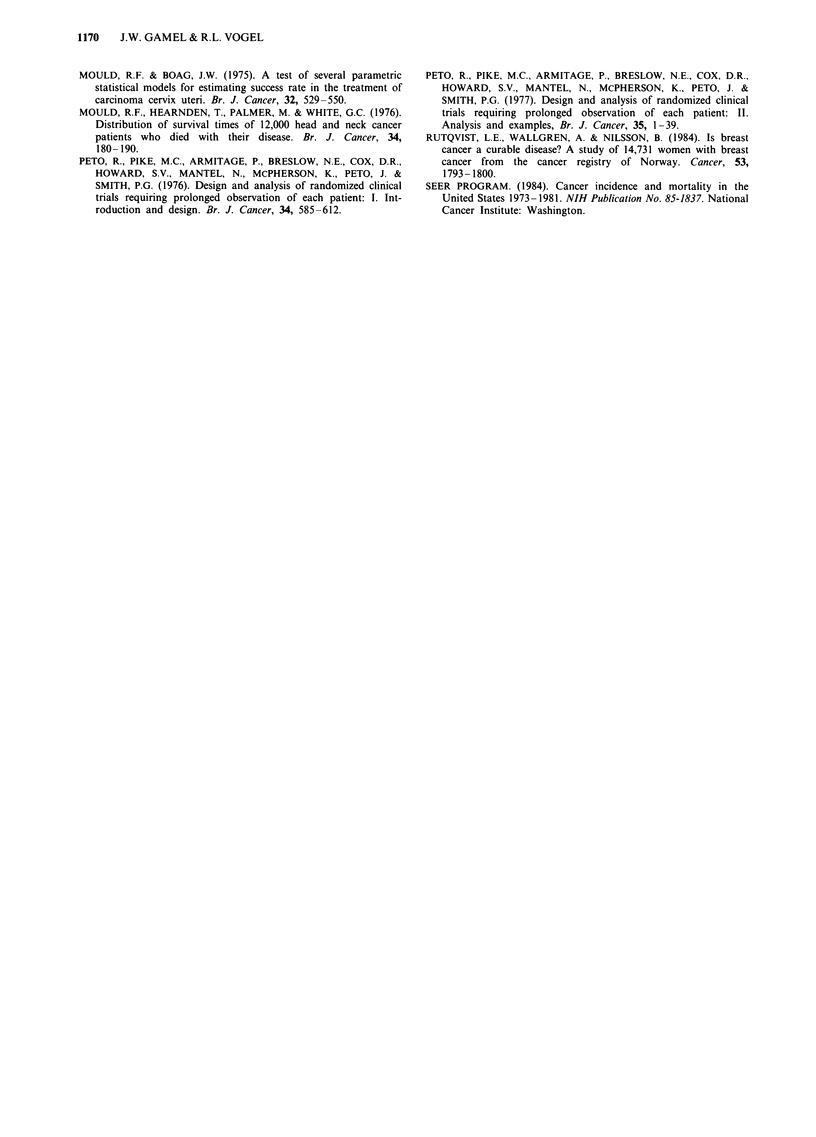


## References

[OCR_00436] Cuzick J., Stewart H., Peto R., Baum M., Fisher B., Host H., Lythgoe J. P., Ribeiro G., Scheurlen H., Wallgren A. (1987). Overview of randomized trials of postoperative adjuvant radiotherapy in breast cancer.. Cancer Treat Rep.

[OCR_00451] Gamel J. W., McLean I. W., Rosenberg S. H. (1990). Proportion cured and mean log survival time as functions of tumour size.. Stat Med.

[OCR_00456] Gamel J. W., Vogel R. L., McLean I. W. (1993). Assessing the impact of adjuvant therapy on cure rate for stage 2 breast carcinoma.. Br J Cancer.

[OCR_00463] Mould R. F., Boag J. W. (1975). A test of several parametic statistical models for estimating success rate in the treatment of carcinoma cervix uteri.. Br J Cancer.

[OCR_00468] Mould R. F., Hearnden T., Palmer M., White G. C. (1976). Distribution of survival times of 12,000 head and neck cancer patients who died with their disease.. Br J Cancer.

[OCR_00474] Peto R., Pike M. C., Armitage P., Breslow N. E., Cox D. R., Howard S. V., Mantel N., McPherson K., Peto J., Smith P. G. (1976). Design and analysis of randomized clinical trials requiring prolonged observation of each patient. I. Introduction and design.. Br J Cancer.

[OCR_00481] Peto R., Pike M. C., Armitage P., Breslow N. E., Cox D. R., Howard S. V., Mantel N., McPherson K., Peto J., Smith P. G. (1977). Design and analysis of randomized clinical trials requiring prolonged observation of each patient. II. analysis and examples.. Br J Cancer.

[OCR_00488] Rutqvist L. E., Wallgren A., Nilsson B. (1984). Is breast cancer a curable disease? A study of 14,731 women with breast cancer from the Cancer Registry of Norway.. Cancer.

